# Ambulatory monitoring unmasks hypertension among kidney transplant patients: single center experience and review of the literature

**DOI:** 10.1186/s12882-019-1442-7

**Published:** 2019-07-27

**Authors:** Eitan Gluskin, Keren Tzukert, Irit Mor-Yosef Levi, Olga Gotsman, Itamar Sagiv, Roy Abel, Aharon Bloch, Dvorah Rubinger, Michal Aharon, Michal Dranitzki Elhalel, Iddo Z. Ben-Dov

**Affiliations:** Department of Nephrology and Hypertension, Hadassah – Hebrew University Medical Center, Jerusalem, Israel

**Keywords:** Ambulatory blood pressure monitoring, Kidney transplantation, calcineurin inhibitors, cyclosporine, tacrolimus, Masked hypertension, Non-dipping

## Abstract

**Background:**

Disagreements between clinic and ambulatory blood pressure (BP) measurements are well-described in the general population. Though hypertension is frequent in renal transplant recipients, only a few studies address the clinic-ambulatory discordance in this population. We aimed to describe the difference between clinic and ambulatory BP in kidney transplant patients at our institution.

**Methods:**

We compared the clinic and ambulatory BP of 76 adult recipients of a kidney allograft followed at our transplant center and investigated the difference between these methods, considering confounding by demographic and clinical variables.

**Results:**

Clinic systolic BP (SBP) and diastolic BP (DBP) were 128 ± 13/79 ± 9 mmHg. Awake SBP and DBP were 147 ± 18/85 ± 10 mmHg. The clinic-minus-awake SBP and DBP differences were − 18 and − 6 mmHg, respectively. The negative clinic-awake ΔSBP was more pronounced at age > 60 years (*p* = 0.026) and with tacrolimus use compared to cyclosporine (*p* = 0.046). Sleep SBP and DBP were 139 ± 21/78 ± 11 mmHg. A non-dipping sleep BP pattern was noted in 73% of patients and was associated with tacrolimus use (*p* = 0.020).

**Conclusions:**

Our findings suggest pervasive underestimation of BP when measured in the kidney transplant clinic, emphasizes the high frequency of a non-dipping pattern in this population and calls for liberal use of ambulatory BP monitoring to detect and manage hypertension.

**Electronic supplementary material:**

The online version of this article (10.1186/s12882-019-1442-7) contains supplementary material, which is available to authorized users.

## Background

### Hypertension in renal transplant recipients

In kidney transplant recipients, hypertension (HTN) is an independent risk factor for cardiovascular diseases (CVD) [1, 2], death [3] and graft malfunction independent of death [3–7], and its prevalence is 50–90% [2, 8, 9]. However, there are no randomized control trials to define target BP levels in renal transplant recipients [10]. The Kidney Disease Improving Global Outcomes (KDIGO) [11] and the American College of Cardiology (ACC) and American Heart Association (AHA) [12] recommendations are to target clinic BP of kidney transplant recipients to levels lower than 130/80 mmHg (a 2C-grade recommendation according to KDIGO). Yet, the recommendations of the National Kidney Foundation are to adjust individually BP levels due to lack in evidence based recommendations [13], while the recent European Society of Hypertension (ESH) and the European Society of Cardiology (ESC) guidelines [14] suggest that the target BP range for patients with chronic kidney disease (CKD) should be 130–139 mmHg, and the Eighth Joint National Committee (JNC 8) recommended values of less than 140/90 mmHg in all CKD patients, not excluding transplant recipients [15].

### ABPM and kidney transplantation

In the general population, clinic BP measurement often differs from ambulatory BP monitoring (ABPM) [16]. Ambulatory BP is monitored at the patient’s ordinary environment, and the measurements are recorded every 20–30 min during a period of 24 h and capture sleep. Hence, ABPM is considered a more reliable measure of BP [17]. Importantly, in the general population ABPM correlates better than clinic BP with end organ damage and cardiovascular outcomes [18–20].

In addition to measuring mean BP, ABPM reveals important data on other values, including awake period BP, asleep period BP, BP variability, morning surge and the sleep-related dipping pattern [21]. Furthermore, it can frame differences between clinic BP measurements and home BP monitoring, by establishing masked hypertension and white-coat hypertension [21, 22]. While white coat hypertension leads to overtreatment [23], masked hypertension represents under-diagnosis and therefore under-treatment [24]. Hence, there is a need in parameters in the daily clinical practice that will help in identifying patients with masked hypertension or a prominent masking effect [25].

Only a few studies have examined the concordance between clinic BP and ABPM in kidney transplant recipients. Though some found a high rate of white-coat hypertension [26–29], others found higher rates of masked hypertension [30–32] in this population. Differences in hypertension definitions can account for some of the discrepancy between the studies. Significantly, ABPM better predicts renal graft function [7].

A non-dipping BP pattern (defined as a < 10% reduction in asleep period BP relative to awake period BP) was reported in up to 79% of the patients with kidney allografts [7, 28, 29]. In the general population, a non-dipping pattern is associated with more complications, including higher rates of stroke, dementia, left ventricular hypertrophy, microalbuminuria and increased carotid intima-media thickening [9, 33]. In kidney transplantation, a non-dipping pattern links with poor allograft function and high Doppler resistive index [34].

The lack of well-established data with regards to diagnosis, treatment and monitoring of hypertension in the renal transplant population gave rise to a contemporary “Call to Action” published by several European scientific societies, encouraging performance of surveys aimed at assessing the prevalence of hypertension (including white-coat and masked hypertension) [35]. Accordingly, in this study, our objectives were to describe clinic and ambulatory BP, dipping patterns and clinic-awake differences in kidney transplant patients and identify associated clinical parameters.

## Methods

### Study population

We enrolled patients post kidney transplantation followed-up in our renal transplant clinic. Inclusion criteria were age 18 years and above and a period of at least 3 months from the transplantation. Pregnant women were not included. Renal transplant physicians recruited potential participants during regular clinic visits. The Helsinki Committee of the Hadassah Medical Organization approved the protocol for this study. All volunteers provided written informed consent prior to participation.

### Data collection

We collected baseline data including age, weight, transplantation details, underlying kidney disease, medications and BP measurements from medical files, and height and missing information directly from the patients. We calculated the aggregate dose of antihypertensive medications as the sum of percentages of each drug’s dose from its maximal dose. We quantified urinary protein excretion and creatinine clearance from a 24-h urine collected during a period before or after the monitoring (up to 3 months for creatinine clearance; for urinary protein, we used data from up to 4 months for renal transplant recipients who were transplanted more than 1 year before the monitoring and 2 months for recipients who were transplanted up to 1 year before the monitoring). Hemoglobin and serum creatinine were measured in the day of monitoring in most patients; in 10 patients, they were measured up to 1 month before or after the monitoring. In a single patient, we collected data from a visit that occurred 4 months before the ABPM. We estimated glomerular filtration rate (eGFR) using the Chronic Kidney Disease Epidemiology Collaboration (CKD-EPI) equation. Trough levels of tacrolimus, everolimus, sirolimus and cyclosporine were measured in the morning of monitoring, before taking the morning dose. In five patients, plasma levels were measured within 8 days after the monitoring; in ten patients, they were measured up to 14 days before the monitoring; in a single patient it was measured 4 months before the monitoring, and in two patients, plasma levels are missing. We make use of information from our institution’s ABPM dataset of referred patients for perspective and comparison with this study’s patients (registry control) [36]. This registry includes patients (47% women) referred predominantly by the primary physicians for accepted clinical indications. Three percent had diabetes and 65% had hypertension (as defined by their ambulatory awake BP).

### Clinic BP measurements

BP was measured *either* by a nurse (observed) using an oscillometric device (Welch Allyn, 52000 series) before entering the physician’s office (*n* = 16), *or* by the physician, using aneroid auscultatory sphygmomanometry (Accucare BP wall mount sphygmomanometer), at least 5 min after arrival at the clinic (*n* = 60). Typically, single measurements were performed. Our study protocol did not address the particulars of clinic measurements, as our aim was to compare real-life clinic measurements with standardized ambulatory monitoring. Measurements were done on an arm without an arterio-venous fistula, while it rested on a table. Clinic BP was calculated as an average of 2–3 visits during the 3 months before conducting ABPM. If only one measurement was performed in this period, an additional measurement taken 3–12 months before the ABPM was included in the average clinic BP. Hypertension medications were in general not changed during this period. However, adherence and as needed use were not systematically assessed.

### Ambulatory blood pressure monitoring

Ambulatory BP was monitored with Oscar2 devices (SunTech Medical). Three adult cuff sizes were available. Measurements were recorded every 20 min during the day and every 30 min during nighttime sleep (according to the participant’s projection) over a 24-h monitoring period. In the ABPM analysis, awake period limits and asleep period limits were further defined according to the participant’s log. Participants with at least 20 awake period measurements and 8 sleep measurements were considered to have a valid ABPM recording. One participant discontinued the monitoring before sleep; his 20 awake period records were included in the study. ABPMs were processed with AccuWin Pro v3 (SunTech Medical).

### Outcome definitions

Clinic HTN was defined as mean clinic SBP ≥ 140 mmHg and/or mean clinic DBP ≥ 90 mmHg, according to JNC 8 report [15]. Awake period HTN (ABPM) was defined as mean SBP ≥ 135 mmHg and/or mean DBP ≥ 85 mmHg; asleep period HTN was defined as SBP ≥ 120 mmHg and/or DBP ≥ 70 mmHg; and 24 h HTN was defined as SBP ≥ 130 mmHg and/or DBP ≥ 80 mmHg, according to the ESC recommendations [37].

The primary outcomes of this study were the differences between clinic SBP/DBP and ambulatory awake period SBP/DBP. The secondary outcomes were prevalence of clinic HTN, awake period ambulatory HTN, asleep period ambulatory HTN; white-coat HTN (defined as clinic HTN without awake period ambulatory HTN); masked HTN (defined as awake period ambulatory HTN without clinic HTN); isolated nocturnal HTN; non-dipping pattern (defined as < 10% reduction in SBP from awake period to sleep time) and a reverse-dipping pattern (defined as higher sleep SBP than awake SBP); as well as assessment of awake and sleep periods’ SBP and DBP variability (standard deviation, SD), and average awake period and sleep period heart rates.

### Statistical analyses

Sample size calculation for our study was driven by the main hypothesis, that in kidney transplantation, clinically meaningful differences exist between office and ambulatory blood pressure. We estimated that roughly 80 patients are required to detect such differences with 80–85% power. Patients’ characteristics are summarized as percentages, means and standard deviations or medians and 1st-3rd quartile values, as appropriate. 95% confidence intervals (CI) for the means of clinic-awake BP differences were calculated using quantile of t-distribution (R package ‘Publish’). We evaluated associations between clinic and ambulatory BP means or indices (e.g. dipping, variability) and demographic and clinical characteristics with general linear (and logistic) regression models using the R statistical environment (and packages including ‘Rmisc’ and ‘nlme’). Proteinuria (mg/d) was log-transformed prior to inclusion in models. Plots were generated using R base functions and the ‘ggplot2’, ‘BlandAltmanLeh’ and ‘waffle’ packages. Two-tailed *P*-values < 0.05 were considered statistically significant. P-value adjustment for multiple comparisons and post-hoc analyses was done by the Benjamini & Hochberg method.

## Results

Between 12/2016 and 2/2018 seventy-six subjects were recruited to the study. Their demographic, anthropometric and clinical characteristics appear in Table 1. Immunosuppressive medication treatment data are displayed in Table 2.

**Table 1:** Characteristics of 76 study participants

Characteristic	Value
Age, years	52 ± 14
BMI, kg/m^2^	26.8 ± 5.2
Diabetes	26%
Smoking, never/former/current	83% / 12% / 5%
Type of allograft, alive	74%
Time since transplantation, years	9.4 (IQR 4.0–16.0)
eGFR (CKD-EPI), ml/min/1.73m^2^	62.2 (IQR 40.7–75.6)
Creatinine clearance, ml/min	61.7 (IQR 42.6–89.0)
Urine protein excretion, mg/d	210 (IQR 140–518)
Hemoglobin, g/dl	13.1 (IQR 11.8–14.3)
Anti-hypertensive medications (any)	74%
ACEi or ARBs	50%
Beta blockers	42%
Calcium channel blockers	39%
Diuretics	17%
Alpha blockers	11%
Other anti-hypertensives	1%
No. of antihypertensive medications	1 (IQR 0–2)
Aspirin	26%
Statins	42%

*Abbreviations*: *BMI* Body mass index, *eGFR* Estimated glomerular filtration rate, *CKD-EPI* Chronic Kidney Disease Epidemiology Collaboration, *ACEi* Angiotensin converting enzyme inhibitor, *ARBs* Angiotensin receptor blocker, *IQR* 1st-3rd quartiles

**Table 2:** Immunosuppressive medication usage among study participants

Medication	Proportion	Daily dose, mg	Levels, ng/ml
Prednisone	93%	5 (5–5)	
Mycophenolate	78%	1000 (625–1500)	
Azathioprine	17%	50 (50–75)	
Cyclosporine	18%	150 (100–150)	61 (48–89)
Tacrolimus	72%	3.0 (2.0–4.25)	7.0 (5.5–8.8)
Everolimus	5%	2.0 (1.875–3.0)	4.2 (3.4–5.2)
Sirolimus	1% (single pt.)	1.6	7.0

Dosage and levels are expressed as median (1st-3rd quartiles)

BP average across the 2–3 most recent clinic visits was 128 ± 13/79 ± 9 mmHg, Table 3. Associations of BP levels with clinical variables are depicted in Table 4 and further described in Additional file 1. Categorically, presence of clinic HTN (SBP ≥ 140 mmHg and/or DBP ≥ 90 mmHg), was noted in 16/56 (29%) of patients treated with antihypertensive medications and in 5/20 (25%) of untreated patients (all 5 also had ambulatory HTN) (see Table 4 and Additional file 1).

**Table 3:** Summary of clinic and ambulatory BP monitoring measurements

Variable	Systolic BP	Diastolic BP	Heart rate
Clinic measurements	128 ± 13	79 ± 9	n/a
24 h period, mmHg or bpm	145 ± 18	83 ± 10	n/a
Awake period, mmHg or bpm	147 ± 18	85 ± 10	78 ± 12
Sleep period, mmHg or bpm	139 ± 21	78 ± 11	67 ± 10
Sleep-related dip, %	5.7 ± 8.0	8.7 ± 9.2	12.5 ± 8.6
Awake period SD, mmHg	15 ± 4	10 ± 3	n/a
Asleep period SD, mmHg	13 ± 5	10 ± 4	n/a

*Abbreviations*: *BP* Blood pressure, *SD* Standard deviation, *BP* Blood pressure, *n/a* Not available

**Table 4:** Associations of clinic and ambulatory BP parameters with clinical variables

BP variable →	Clinic	Clinic	Awake	Sleep	Sleep dip	Sleep	Non-dip	Awake SD
Clinical variable ↓	SBP/DBP	HTN	SBP/DBP	SBP/DBP	SBP/DBP	HTN	SBP/DBP	SBP/DBP
Age > 60 y	NS/−4.6^a^	–	+ 10.0^a^/NS	–	–	–	–	+ 3.1^b^/NS
Sex, male	–	–	–	–	–	–	–	–
BMI, kg/m^2^	+ 0.82^b^/NS	–	–	–	–	–	–	–
Diabetes	+ 7.9^a^/NS	–	NS/−6.3^a^	+ 11.3^a^/NS	NS/−0.07^b^	–	–	+ 2.4^a^/NS
Cadaveric allograft	–	–	NS/−6.3^a^	–	–	–	–	–
Time since Tx, y	–	–	–	–	–	− 0.10^a^	–	–
Proteinuria, log_10_(mg/d)	+ 9.8^b^/NS	+ 1.8^b^	+ 10.0^a^/NS	–	–	–	–	–
HTN medications, #	+ 3.1^c^/NS	–	+ 3.4^a^/NS	+ 4.0^a^/NS	–	–	–	–
HTN medications, cumulative dose	+ 0.05^c^/NS	–	+ 0.07^c^/NS	0.07^b^/NS	–	–	–	–
HTN medications, evening dosing	–	–	–	–	–	–	–	NS/−1.2^a^
eGFR, ml/min/1.73m^2^	−0.22^c^/NS	− 0.03^a^	–	–	–	–	–	–
CrCl, ml/min	−0.12^a^/NS	− 0.03^a^	–	–	–	–	–	–
P. creatinine, μmol/l	–	+ 0.01^a^	–	–	–	–	–	–
Tacrolimus vs CsA	–	–	–	–	NS/−0.08^b^	+ 1.9^a^	+ 1.5^a^/NS	–
Hemoglobin, g/dl	–	–	–	–	+ 0.01^a^/+ 0.01^a^	–	–	–

Cell values represent the coefficient (ß) in a model with the BP-related parameter (column names) as the independent variable and the clinical parameter (row names) as the dependent variable. *Abbreviations*: *BMI* Body mass index, *eGFR* Estimated glomerular filtration rate, *CrCl* Creatinine clearance, *CsA* Cyclosporine, *SBP* Systolic blood pressure, *DBP* Diastolic blood pressure, *HTN* Hypertension, *SD* Standard deviation, *NS* (as well as ‘-’) Non-significant; ^a^p < 0.05 (but not significant after adjustment for multiple comparisons); ^b^p ≤ 0.005 (in some cases borderline after adjustment for multiple comparisons; ^c^*p* ≤ 0.001 – see multiple comparison-adjusted p-values in Additional file 3

Awake, sleep and 24 h BP as well as sleep-related dipping averages are displayed in Table 3. Links with clinical parameters are depicted in Table 4 and in Additional files 1 and 2. Presence of awake HTN (average SBP ≥ 135 mmHg and/or average DBP ≥ 85 mmHg) was noted in 43/56 (77%) of patients treated with antihypertensive medications and in 16/20 (80%) of untreated patients. Sleep HTN (average SBP ≥ 120 mmHg and/or average DBP ≥ 70 mmHg) was noted in 48/55 (87%) of patients treated with antihypertensive medications and in 16/20 (80%) of untreated patients.

Isolated nocturnal HTN was present in 11% of participants. Rates of normal systolic BP dipping pattern, non-dipping pattern and reverse dipping pattern were 26.7% (20 patients), 53.3% (40 patients) and 20% (15 patients), respectively. In contrast, non-dipping of heart rate (< 10% reduction during sleep) was noted in only 43% of subjects.

**Fig. 1:**
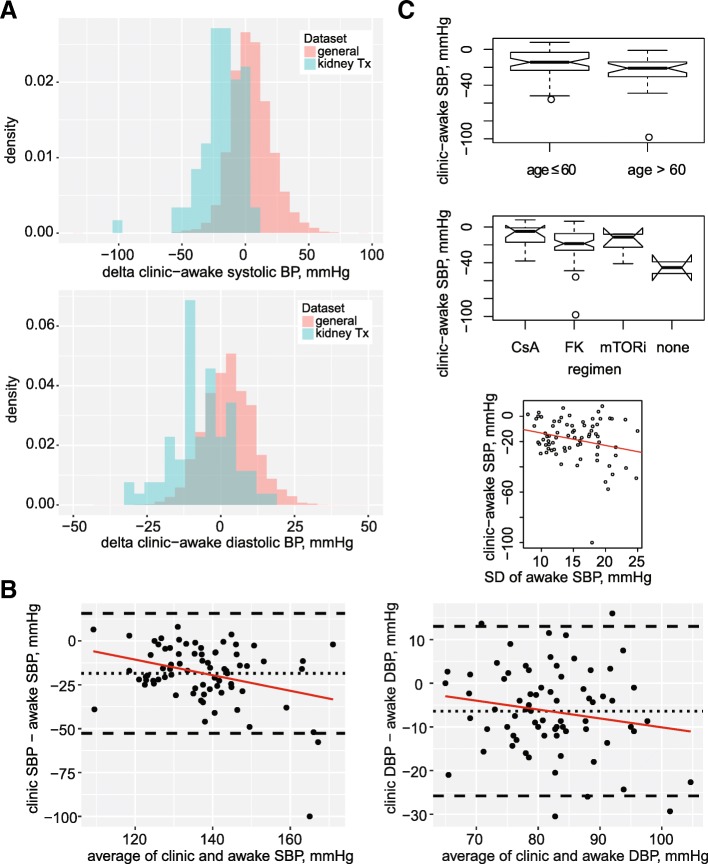
Distribution and clinical correlates of the clinic-awake BP difference. **a** Density histograms plotting the distribution of ΔBP values; clinic-awake systolic BP (top) and diastolic BP (bottom). For reference, the respective distributions are shown from an ABPM referral dataset. **b** Bland-Altman plots of ΔSBP (left) or ΔDBP (right) vs. the respective average of awake and clinic BP. The dotted line represents the mean difference while the dashed lines represent the 95% limits of agreement (±2 standard deviations of the difference) (**c**) Dependence of ΔSBP on age (top), immuno-suppressive regimen (middle) and the variability of awake SBP (bottom)

The discrepancy between ambulatory and clinic BP was expressed as clinic minus awake SBP, ΔSBP, and clinic minus awake DBP, ΔDBP. The distribution of ΔSBP (− 18.5, 95% CI − 22.4 to − 14.6 mmHg) and ΔDBP (− 6.4, 95% CI − 8.6 to − 4.2 mmHg) values is shown in Fig. 1a, alongside analogous control data from an independent dataset of referred patients [36] for perspective. ΔSBP and ΔDBP are also shown versus the respective average values of awake and clinic BP (Bland-Altman plots, Fig. 1b). The standard deviations (SD) of the differences were 17.1 and 9.7 mmHg respectively, and the 95% limits of agreement (1.96*SD) were − 52.6 to + 15.7 mmHg for SBP and − 35.8 to + 13.0 for DBP (the limits of agreement show the estimated range within which the differences between single readings by the two modalities would fall on 95% of occasions) (Fig. 1b). ΔSBP was positively dependent on age (mean ΔSBP for age > 60 –24.7, 95% CI − 32.9 to − 16.5 mmHg; mean ΔSBP for age ≤ 60 –15.4, 95% CI − 19.6 to − 11.3 mmHg; *p* = 0.026), on the immunosuppressive regimen (mean ΔSBP for cyclosporine − 9.6, 95% CI − 17.4 to − 1.9 mmHg; mean ΔSBP for tacrolimus use − 19.8, 95% CI − 24.4 to − 15.1 mmHg; mean ΔSBP for no calcineurin inhibitor use − 26.0, 95% CI − 42.7 to − 9.3 mmHg; *p* = 0.025) and on the variability of awake systolic BP (*p* = 0.034) (Fig. 1c and Table 5). After adjustment for age > 60, the association of ΔSBP with the immunosuppressive regimen dampened (*p* = 0.094), as did the dependence on age as a continuous variable (*p* = 0.064). The association of ΔSBP with the immunosuppressive regimen did not lose significance after adjustment for time since transplantation (*p* = 0.048) or for diabetes (*p* = 0.046).

**Table 5:** Associations of the clinic-awake BP differences (ΔBP) with clinical variables

Parameter	ΔSBP		ΔDBP	
	Coefficient	*P*-value	Coefficient	*P*-value
Age > 60 years	**−9.2**	**0.026**	− 1.2	0.624
BMI, kg/m^2^	0.3	0.402	−0.1	0.722
Diabetes	0.2	0.964	3.0	0.231
Past smoking vs. never	−0.5	0.929	−4.7	0.179
Type of allograft, cadaveric vs. alive	0.7	0.874	2.5	0.324
Time since transplantation, years	0.1	0.614	−0.03	0.809
eGFR (CKD-EPI), ml/min/1.73m^2^	−0.1	0.171	0.01	0.855
Creatinine clearance, ml/min	−0.1	0.302	0.02	0.525
Urine protein excretion, mg/d (log_10_)	−0.3	0.956	2.4	0.364
Hemoglobin, g/dl	−1.3	0.244	0.2	0.697
Anti-hypertensive medications (any)	6.1	0.173	1.6	0.532
ACEi or ARBs	3.4	0.393	−0.2	0.915
Beta blockers	0.8	0.847	2.1	0.358
Calcium channel blockers	−0.2	0.968	0.2	0.947
Diuretics	−3.4	0.521	−2.7	0.365
Alpha blockers	−2.3	0.727	0.9	0.815
No. of antihypertensive medications	−0.3	0.800	−0.2	0.798
Aspirin	−0.1	0.984	3.0	0.242
Tacrolimus vs. cyclosporine	**−10.1**	**0.046**	−1.8	0.546
Non-CNI vs. cyclosporine	**−16.3**	**0.038**	−3.5	0.446
Siesta	4.7	0.441	0.5	0.892
Oscillometric vs. aneroid clinic measurement	7.0	0.146	3.7	0.177
Clinic BP, mmHg	**0.4**	**0.003**	**0.4**	**< 0.001**
SD of awake ambulatory BP, mmHg	**−1.0**	**0.034**	−0.7	0.076

*Abbreviations*: *BMI* Body mass index, *eGFR* Estimated glomerular filtration rate, *CKD-EPI* Chronic Kidney Disease Epidemiology Collaboration, *ACEi* Angiotensin converting enzyme inhibitor, *ARBs* Angiotensin receptor blocker, *CNI* Calcineurin inhibitor, *BP* Blood pressure, *SD* Standard deviation

Bold entries are significant

**Fig. 2:**
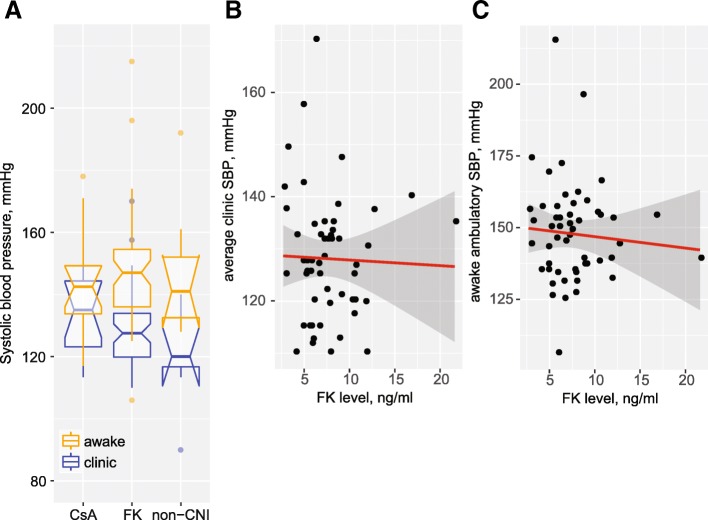
Clinic and awake systolic BP in relation to tacrolimus use. **a** Box plots summarizing clinic and awake SBP levels among patients treated with cyclosporine, tacrolimus or neither calcineurin inhibitor. **b**, **c** Lack of relationship was noted between clinic SBP (**b**) or ambulatory awake SBP (**c**) and tacrolimus trough levels

Figure 2a shows clinic and ambulatory awake systolic BP values, in an overlapping manner, according the immunosuppressive regimen. The discrepancy appears wider among patients receiving tacrolimus. However, neither clinic (Fig. 2b) nor awake (Fig. 2c) systolic BP averages were dependent on trough drug levels among patients receiving tacrolimus.

**Fig. 3:**
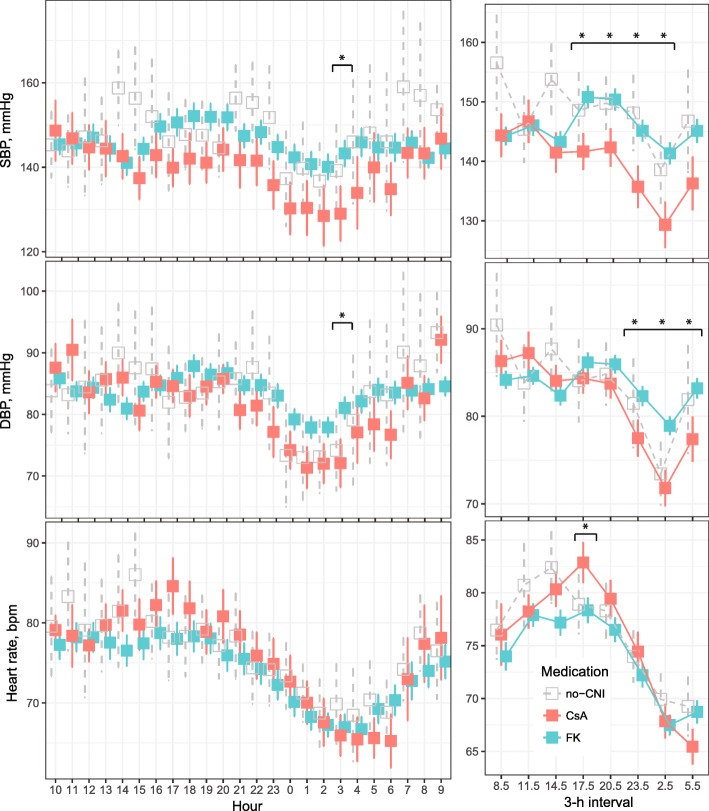
Diurnal ambulatory monitoring patterns in relation to calcineurin inhibitor use. Hourly averages (left panels) and 3-h interval averages (right panels) of ambulatory systolic BP (top), diastolic BP (middle) and heart rate (bottom) are plotted according to calcineurin inhibitor use, with bars indicating standard error of the mean. Asterisks (*) denote time points or intervals in which levels were significantly different between tacrolimus and cyclosporine-treated study patients

To further characterize diurnal BP patterns in relation to calcineurin inhibitor use we extracted individual patients’ BP measurements from all ABPM tracings. Hourly averages as well as 3 h interval averages are plotted according to calcineurin inhibitor use in Fig. 3. SBP was significantly higher among tacrolimus users in the 16:00–04:00 time intervals; DBP was higher throughout the 22:00–07:00 time intervals; and heart rate was significantly lower among tacrolimus users in the 16:00–19:00 time interval. According to a linear mixed effects model, the diurnal time interval was a significant predictor of SBP, DBP and heart rate (all *p*-values< 0.0001), while the interaction between medication and time interval predicted SBP (*p* = 0.061) and DBP (*p* = 0.013).

**Fig. 4:**
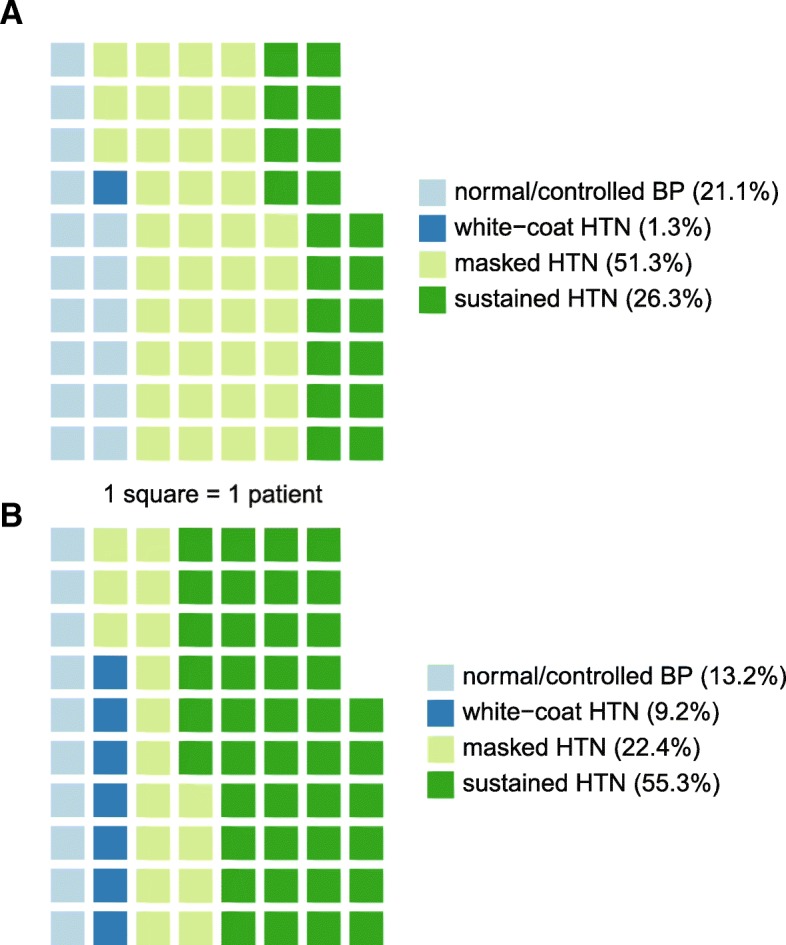
Rates of clinic vs awake blood pressure patterns. Waffle plots showing the prevalence of blood pressure control patterns among study patients, considering (**a**) JNC8 clinic hypertension cutoffs, 140/90 mmHg, or (**b**) KDIGO and ACC-recommended clinic cutoffs, 130/80 mmHg

According to JNC8 definitions for clinic HTN and to ESC-ESH recommendations for ambulatory HTN, rates of white coat HTN, masked HTN, sustained HTN and normotension were 1.3, 51.3, 26.3, 21.1%, respectively (Fig. 4a). However, according to KDIGO and ACC recommendations for clinic BP targets (< 130/80 mmHg), the respective proportions were 9.2, 22.4, 55.3, and 13.2% (Fig. 4b).

## Discussion

In our study, employing ambulatory blood pressure monitoring, we found that patients followed at our kidney transplant clinic have appreciably higher systolic and diastolic BP levels outside the office as compared to their clinic measurements. The differences, averaging 18 and 6 mmHg, respectively, expose widespread underestimation of BP, misclassification of BP control and masked hypertension in our kidney transplantation clinic. The limits of agreement between ambulatory awake and clinic BP measurements were wide (see Fig. 1b).

In our study, the negative clinic–awake SBP difference (i.e., masking) was more pronounced with age over 60 years and with tacrolimus use (versus cyclosporine). In the general population, one study found age to be a risk factor for masked HTN [38]; other studies, however, found that the clinic-awake difference increases (i.e., more positive) with age [25] and thus elderly have more white-coat effect [39]. Tacrolimus use was found in other studies [40, 41] in association with lower clinic BP. We suggest that this association may not be true with regards to ambulatory BP and may thus lead to underestimation (and under-treatment) of BP in tacrolimus-treated patients evaluated using clinic measurements alone. However, we have not pre-specified subset analyses based on age and calcineurin inhibitor regimen, and patients receiving cyclosporine were scarce, and thus results are not conclusive.

As a result of the negative clinic-awake BP difference in our patients, masked HTN was uncovered much more commonly than white coat HTN. This finding is sensitive to clinic HTN definitions. The ACC/AHA and KDIGO-adopted lower clinic BP thresholds amplify the prevalence of sustained HTN on account of masked HTN and that of white coat HTN on account of normotension. Overall, KDIGO/ACC/AHA guideline cutoffs lead to more agreement between clinic- and ABPM awake-based determinations than JNC 8 cutoffs (69% vs. 47%).

### Review of the literature with regards to ΔBP in kidney transplantation

Previous studies have also shown negative clinic-ambulatory BP differences in renal transplant populations, albeit of lesser extent. However, in several other studies opposing results have been noted. These previous studies differ one from another in patients’ age, clinic BP and ABPM methods and in exclusion criteria (Table 6).

**Table 6:** clinic-awake SBP and DBP differences from previous studies

Study	No.	Age	ΔSBP	ΔDBP	Clinic BP	FK vs. CsA	Exclusion	Other
Ahmed 2015 [30]	98	55	−3.5	−7.2	all study measurements	no comparison	unstable BP	new center
Kayrak 2014 [32]	113	44	−9	−6.4		no comparison	uncontrolled hypertension	
Mallamaci 2016 [42]	172	46	−1	−1	monitoring day only	no comparison^a^		
Mallamaci 2018 [43]	260	47	+ 6	0	after ABPM, mean 3.7 years	no comparison		
Demikrol 2016 [29]	87	38	+ 6	+ 4.5		no difference in dipping	comorbidities	
Wen 2012 [28]	244	53	+ 3.6	+ 2.5	measurement within 5 days	no effect on ABPM values		higher clinic BP levels
David 2014 [26]	49	35	+ 3–12	+ 6–8	clinic and 1st and last ABPM measurements	FK only		
Fresendo 2012 [27]	868	53	+ 7	+ 1	absent data	no comparison		reduced GFR
Paoletti 2009 [7]	94	55	+ 4	+ 1		Lower PP with FK		higher clinic BP levels

*Abbreviations*: *SBP* Systolic blood pressure, *DBP* Diastolic blood pressure, *FK* Tacrolimus, *CsA* Cyclosporine, *BP* Blood pressure, *CNI* Calcineurin inhibitor, *ABPM* Ambulatory blood pressure monitoring, *DM* Diabetes mellitus, *HF* Heart failure, *IHD* Ischemic heart disease; *CMP* Cardiomyopathy, *GFR* Glomerular filtration rate, *PP* Pulse pressure

^a^ Sirolimus associated with higher dipping ratio

In a study of 98 patients from New-Zealand [30], the mean differences between clinic SBP and DBP and average 24 h SBP and DBP were − 3.5 mmHg and − 7.2 mmHg, respectively. These values are also negative, but they are less negative than the current study's values. There are several explanations for this difference: (1) In this study, clinic SBP was compared to 24 h BP, which was lower than awake BP. (2) The clinic BP was measured as an average of all the measurements in the study period, apparently including measurements that were done after (and may have been affected by) the ABPM. (3) The study was conducted at a research center, with which the patients were not familiar. (4) Patients with unstable BP levels (not defined) were not included. In a 113-patient Turkish study [32], the mean differences between clinic SBP and DBP and average awake SBP and DBP were − 9.0 mmHg and − 6.4 mmHg, respectively. Patients with uncontrolled HTN (not defined) were excluded. Lastly, in 172 Italian patients, the clinic – awake SBP and DBP differences were − 1 mmHg [42]; clinic BP was measured only in the monitoring day.

On the other hand, in a 260-patient Italian study, the clinic – awake SBP and DBP differences were + 6 and 0 mmHg, respectively [43]. The clinic measurements in the Italian study were done after the ABPM, a fact that could has influenced the results, and the clinic BP was measured as the mean of BP measurements within a mean period of 3.7 years. In another 87-patient Turkish study, the clinic – awake SBP and DBP differences were + 6 and + 4.5 mmHg, respectively [29]; however, several exclusion criteria have been used in this Turkish study (history of diabetes mellitus, heart failure, ischemic heart disease, cardiomyopathy, or significant valvular heart disease; hemoglobin level < 10 g/dL; and serum creatinine level > 1.5 mg/dL). Among 244 Canadian patients, the clinic – awake SBP and DBP differences were + 3.6 and + 2.5 mmHg, respectively [28]. In this Canadian study, one clinic measurement was recorded, obtained within 5 days of ABPM (not mentioned if before or after the ambulatory monitoring). More importantly, the average clinic BP in the Canadian study was 137/79 mmHg, higher than the value in our study. Higher clinic BP levels are associated with less negative clinic – awake BP differences (Fig. 1b).

In a 49-patient Indian study, the clinic – awake SBP and DBP differences were positive at 2, 4, 6, and 9 months after transplantation [26]; however, the mean age of the participants in the Indian study was 35 years, and the methodology of clinic BP measurement was different (it was calculated as a mean of a clinic measurement, the first ABPM measurement and the last ABPM measurements). In a Spanish multicenter study (868 patients), the clinic – awake SBP and DBP differences were + 7 and + 1 mmHg, respectively [27]; however, kidney transplant recipients with Scr > 2.5 mg/dL or with eGFR < 30 ml/min/1.73m^2^ were not included, and description of clinic and ABPM measurement methodology is lacking. In a 94-patient Italian study, the clinic – awake SBP and DBP differences were + 4 and − 1 mmHg, respectively [7]. A possible explanation for this difference is that in this Italian study the clinic BP was 150/86 mmHg on average, and in our study higher clinic BP measurements associated with less negative delta values, as mentioned previously (Fig. 1b).

As in our study, the tendency for a negative clinic–awake BP difference has been observed in other (non-transplant) CKD populations. In an US cross-sectional study on 1492 CKD patients, the differences between clinic and ambulatory awake SBP and DBP were − 5.9 and − 6.4 mmHg, respectively [44]. In a Japanese study [45], masked HTN was more prevalent than white coat HTN among stage 3–5 CKD patients.

In addition to the clinic-awake discrepancy, nocturnal measurements have revealed that 83% of our patients have sleep HTN. This finding is in line with previous results (69% [42], 71% [30]). Also, only 23% of the patients in our study were normal dippers. This too is similar to previous findings (21% [7], 32% [29], 14% [27], 27% [46]). High rates of non-dipping have also been shown in liver transplant patients, treated with cyclosporine or tacrolimus [47], and in cyclosporine treated heart transplant recipients [48]. In our study, a non-dipping pattern was associated with tacrolimus use, even after adjustment for diabetes, time since transplantation and age. Low hemoglobin levels were linked to lower SBP and DBP dipping ratio; this finding is consistent with data from the general population [49]. As compared with BP dipping, heart rate dipping during sleep, an independent predictor of mortality [50], was relatively preserved in our patients.

In the absence of significant relationships with age, diabetes and BMI, the elevated magnitude of BP masking and non-dipping observed in our patients is possibly a consequence of disturbed volume status, as has been shown for tacrolimus treatment [51], and derangements in autonomic nervous system activity. Also, in as much as anxiety and stress may be involved in the white-coat BP response [52], it is tempting to speculate that for a kidney transplant patient in a routine visit, the kidney transplant clinic constitutes a comforting environment, with familiar fellow patients and more importantly staff, with whom not rarely a patient may have more than a decade long familiarity (being that kidney transplant physicians and nurses also care for dialysis and pre-dialysis patients). Thus, hypertension may be masked in the kidney transplant clinic much more often than a white coat response is elicited.

### Limitations

Recruitment of participants was performed predominantly by their transplant nephrologists, possibly leading to referral biases toward patients who their physicians thought them to have indications for ABPM. Also, clinic BP measurements were not done in a uniform manner: 5 of 7 physicians used aneroid sphygmomanometry, while two relied on oscillometric measurements taken by the clinic nurse. Only one measurement was typically recorded in each visit. Our study has no dedicated control group, although we did use parallel information from our institution’s general ABPM dataset for perspective. Our study’s relatively small size (76 patients who take different antihypertensive medications) is a limitation too, and caution is advised in interpreting some results also due to their post-hoc nature.

On the other hand, our study’s strength is in examining the clinic-awake BP difference as a continuous variable, and therefore independent of hypertension definitions. To our knowledge, it is the first study that describes diurnal-based BP differences between immunosuppressant regimens, thus generating hypothesis for further studies.

## Conclusions

We conclude that ABPM is justified in the renal transplant population. This is due to the high proportion of masked hypertensives and a predominating negative clinic-awake BP difference, and due to the lack of robust predictors of masking.

## Additional files

Additional file 1: Supporting description and correlation analysis of blood pressure parameters. A Microsoft Word file with supplementary results. (DOCX 32 kb)

Additional file 2: PDF file containing 4 supplementary figures. (PDF 440 kb)

Additional file 3: **Table S1.** Benjamini-Hochberg adjusted *p*-values corresponding to the association matrix presented in Table 4 (values < 0.05 are highlighted). A Microsoft Excel file containing supplementary results. (XLSX 12 kb)

## Data Availability

Datasets supporting the conclusions of this article are in part included within the article and its additional files. The complete datasets generated and/or analyzed during the current study are available from the corresponding author on reasonable request.

## Abbreviations

ABPM: Ambulatory blood pressure monitoring
ACC: The American College of Cardiology
BMI: Body mass index
BP: Blood pressure
CKD: Chronic kidney disease
CKD-EPI: Chronic Kidney Disease Epidemiology Collaboration
CVD: Cardiovascular disease
DBP: Diastolic BP
eGFR: Estimated glomerular filtration rate
ESC: European Society of Cardiology
ESH: European Society of Hypertension
HTN: Hypertension
JNC 8: The Eighth Joint National Committee
KDIGO: The Kidney Disease Improving Global Outcome
SBP: Systolic BP
SD: Standard deviation
