# Supplementary material for: Ambulatory monitoring unmasks hypertension among kidney transplant patients: single center experience and review of the literature
Source: BMC Nephrol. 2019 Jul 27;20:284. doi: 10.1186/s12882-019-1442-7 (PMC6661097; doi:10.1186/s12882-019-1442-7)
Supplement: Supplementary file 1 — Supporting description and correlation analysis of blood pressure parameters. A Microsoft Word file with supplementary results. (DOCX 32 kb) [file 12882_2019_1442_MOESM1_ESM.docx]

**Ambulatory monitoring reveals widespread underestimation of blood pressure among kidney transplant patients: Single center experience and review of the literature**

Short title: **ABPM in kidney allograft recipients**

Eitan Gluskin, Keren Tzukert, Irit Mor-Yosef Levi, Olga Gotsman, Itamar Sagiv, Roy Abel, Aharon Bloch, Dvorah Rubinger, Michal Aharon, Michal Elhalel and Iddo Z. Ben-Dov

**Supplementary Results**

**Clinic blood pressure**

Clinic SBP was positively associated with BMI (ß=+0.82, p=0.003), diabetes (ß=+7.9, p=0.017), urinary protein excretion (log_10_) (ß=+9.8, p=0.003), number (ß=+3.1, p=0.001) and the aggregate relative dosage (ß=+0.05, p<0.001) of antihypertensive medications and was negatively associated with eGFR (ß=-0.22, p<0.001) and with creatinine clearance (ß=-0.12, p=0.014). Clinic DBP was inversely linked with age>60 (ß=-4.6, p=0.034) (**Fig.S1**). Neither was dependent on sex, time since transplantation, donor type, smoking or hemoglobin level.

Categorically, clinic HTN (SBP ≥ 140 mmHg and/or DBP ≥ 90 mmHg), was inversely associated with eGFR (ß=-0.03, p=0.020) and creatinine clearance (ß=-0.03, p=0.021), and directly associated with serum creatinine (ß=+0.01, p=0.019) and degree of proteinuria (log_10_) (ß=+1.8, p=0.004) (**Fig.S1**), but no significant relationship was found with age, sex, diabetes (or other aforementioned parameters).

**Ambulatory blood pressure**

Significant positive links were detected between awake SBP and age>60 (ß=+10.0, p=0.018), log_10_ urinary protein excretion (ß=+10.0, p=0.032), and number (ß=+3.4, p=0.011) and relative dosage (ß=+0.07, p=0.001) of antihypertensive medications. Links were *not* detected between awake SBP and BMI (p=0.199) or eGFR (p=0.291). Awake DBP was negatively associated with cadaveric donation (ß=-6.3, p=0.018) and diabetes (ß=-6.3, p=0.018). Sleep SBP was positively associated with diabetes (ß=+11.3, p=0.041), and with the number (ß=+4.0, p=0.016) and cumulative dose (ß=+0.07, p=0.005) of antihypertensive medications (**Fig.S2**).

The SBP dip associated positively with hemoglobin level (ß=+0.01, p=0.014), while he DBP dip negatively associated with diabetes (ß=-0.07, p=0.003) and with the immunosuppressant regimen (tacrolimus vs. cyclosporine; ß=-0.08, p=0.004) and positively related to hemoglobin level (ß=+0.01, p=0.038) (**Fig.S2**).

Presence of awake HTN (average SBP ≥ 135 mmHg and/or average DBP ≥ 85 mmHg) was not dependent on any of the documented clinical variables. Sleep HTN (average SBP ≥ 120 mmHg and/or average DBP ≥ 70 mmHg) was negatively linked with time since transplantation (ß=-0.10, p=0.019) and also associated with the immunosuppressant regimen (tacrolimus vs. cyclosporine; ß=+1.9, p=0.011) (**Fig.S3**).

Non-dipping (<10%) of SBP during sleep positively associated with tacrolimus vs. cyclosporine use (ß=+1.5, p=0.020). Non-dipping of heart rate (<10%) associated with the number of antihypertensive medications (ß=+0.43, p=0.016) (**Fig.S3**).

Awake BP variability (SD of awake BP) was 15.3±4.2 mmHg systolic and 10.1±2.7 mmHg diastolic. It positively associated with age>60 (ß=+3.1, p=0.002) and with diabetes (ß=+2.4, p=0.031). Awake DBP variability was negatively related to evening dosing of antihypertensive medications (ß=-1.2, p=0.043; **Fig.S4**).

**Supplementary figure legends**

**Fig.S1** **Associations between clinical parameters and clinic BP.** The plots are showing clinical parameters found to be associated significantly with clinic systolic BP (top 2 rows), clinic diastolic BP (middle row) or clinic hypertenion, ≥140/90 mmHg (bottom row).

**Fig.S2** **Associations between clinical parameters and ambulatory BP.** The plots are showing clinical parameters found to be associated significantly with awake SBP or DBP, sleep SBP or DBP or the sleep related SBP or DBP dipping magnitude.

**Fig.S3** **Associations between clinical parameters and sleep-related abberations.** The plots are showing clinical parameters found to be associated significantly with sleep hypertension or non-dipping (<10%) of blood pressure or heart rate during sleep.

**Fig.S4** **Associations between clinical parameters and BP variability.** The plots are showing clinical parameters found to be associated significantly with variability (standard deviation) of awake systolic BP.
