# Supplementary material for: Ambulatory monitoring unmasks hypertension among kidney transplant patients: single center experience and review of the literature
Source: BMC Nephrol. 2019 Jul 27;20:284. doi: 10.1186/s12882-019-1442-7 (PMC6661097; doi:10.1186/s12882-019-1442-7)
Supplement: Supplementary file 2 — PDF file containing 4 supplementary figures. (PDF 440 kb) [file 12882_2019_1442_MOESM2_ESM.pdf]

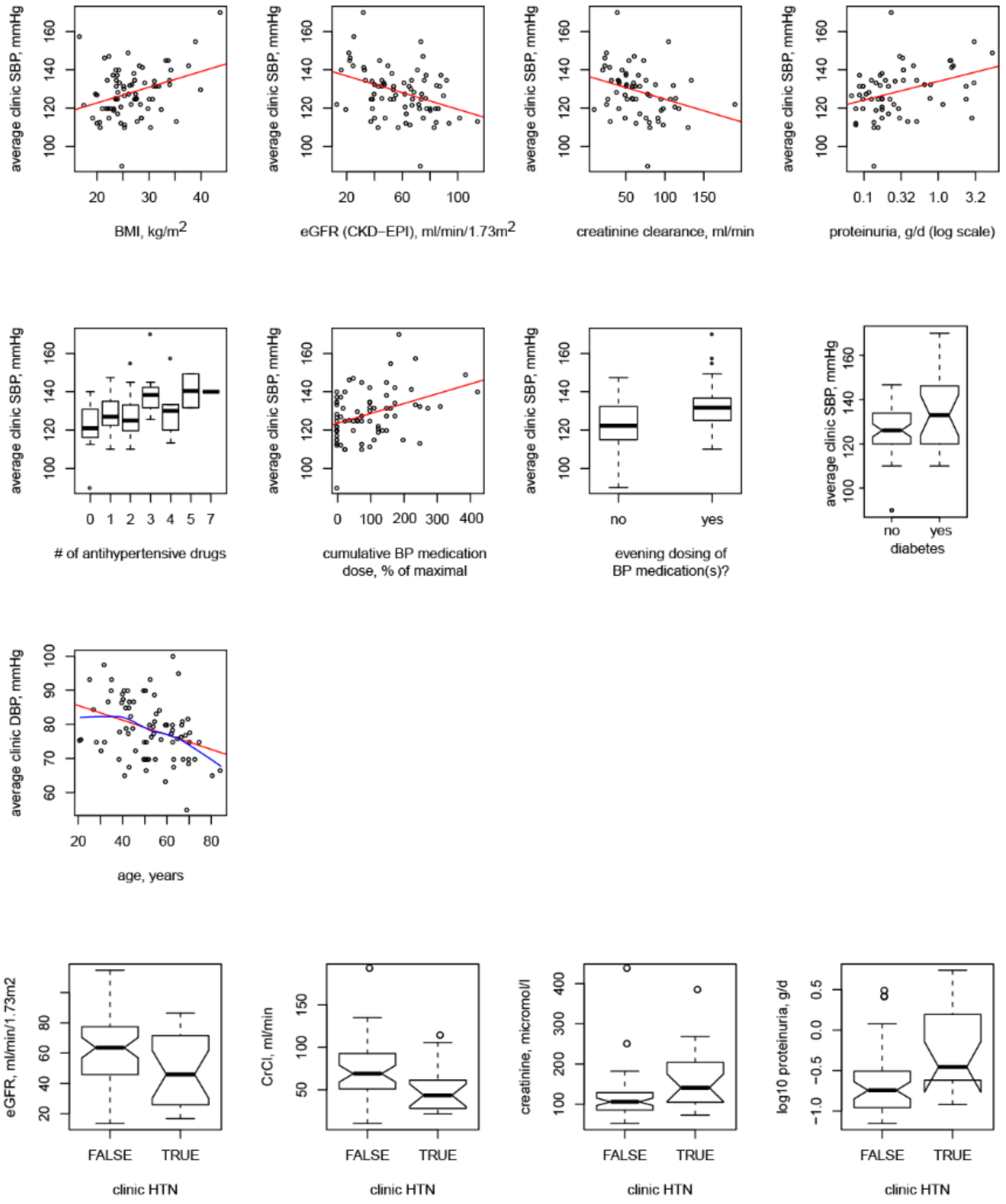

Figure S1: **Associations between clinical parameters and clinic BP.** The plots are showing clinical parameters found to be associated significantly with clinic systolic BP (top 2 rows), clinic diastolic BP (middle row) or clinic hypertension, ≥140/90 mmHg (bottom row).

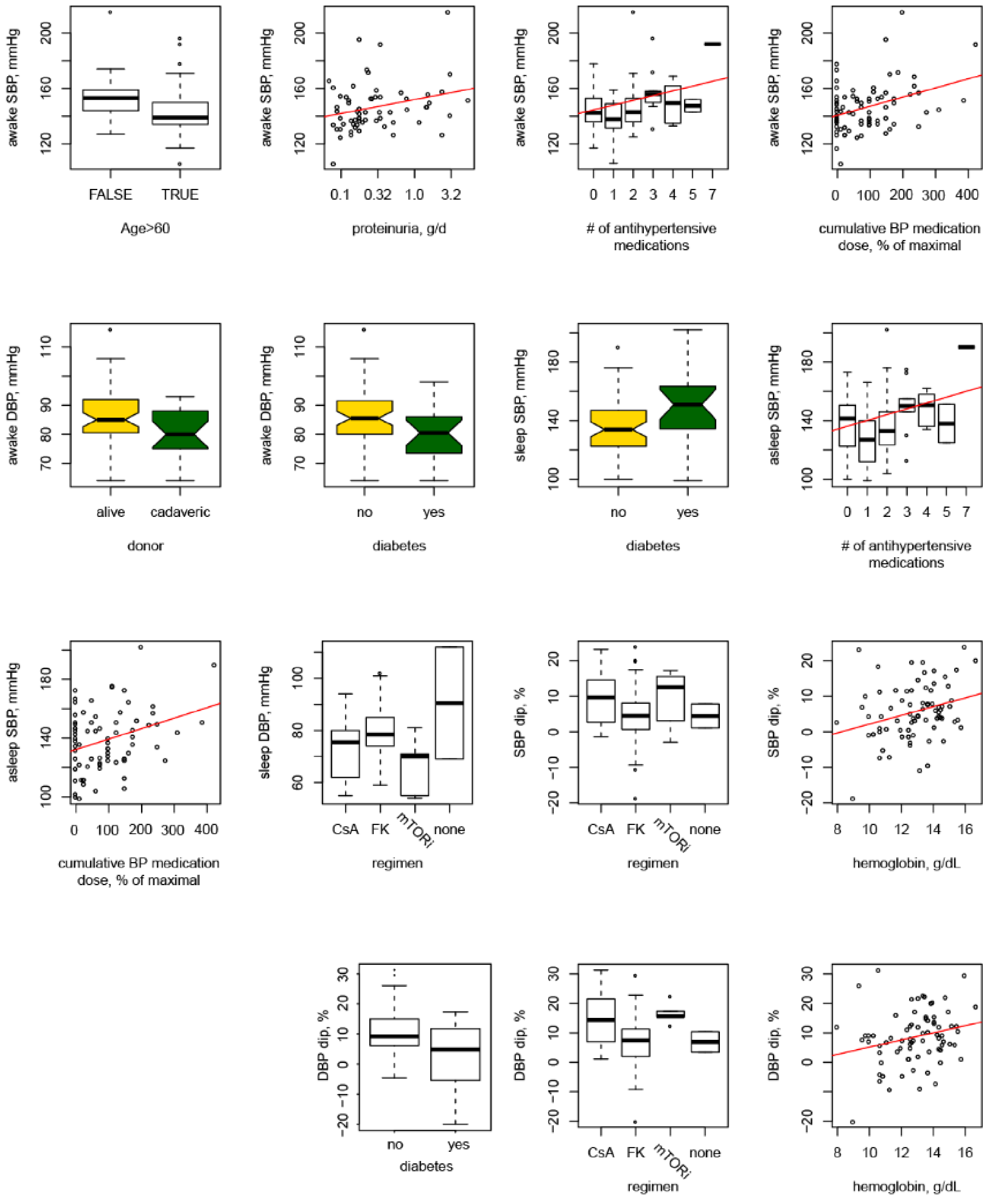

Figure S2: **Associations between clinical parameters and ambulatory BP.** The plots are showing clinical parameters found to be associated significantly with awake SBP or DBP, sleep SBP or DBP or the sleep related SBP or DBP dipping magnitude.

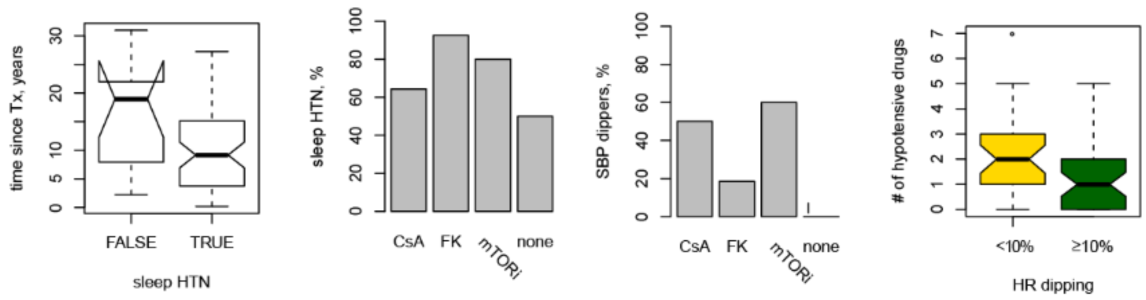

Figure S3: **Associations between clinical parameters and sleep-related abberations.** The plots are showing clinical parameters found to be associated significantly with sleep hypertension or non-dipping (<10%) of blood pressure or heart rate during sleep.

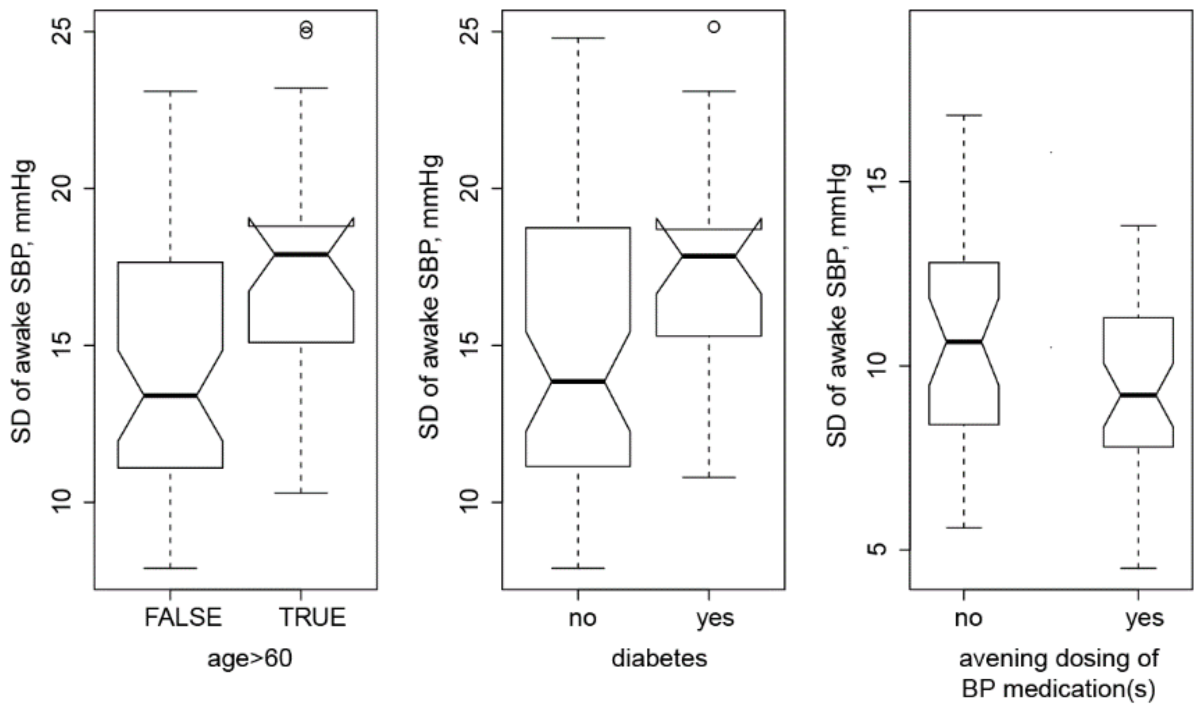

Figure S4: **Associations between clinical parameters and BP variability.** The plots are showing clinical parameters found to be associated significantly with variability (standard deviation) of awake systolic BP.
